# Prothrombin gene mutations do not cause recurrent pregnancy loss in the Indian population

**DOI:** 10.18502/ijrm.v17i5.4605

**Published:** 2019-06-26

##  Dear Editor,

As defined by the American Society of Reproductive Medicine, recurrent miscarriage or recurrent pregnancy loss is defined as two or more failed pregnancies. (1). There are various causes for RPL and yet, in about 40% of cases, no cause can be identified after extensive investigation. It is believed that inherited and acquired thrombophilia like Protein C/S deficiency and genetic mutations in the Factor V and the Prothrombin gene maybe a cause of RPL (2). There is a significant association between the Prothrombin G2021M mutation (PTG) with RPL which has been shown by numerous other studies (3). The data with respect to PTG G20210A mutations and its association with RPL is scant in Indian population.

In this study, we evaluated a total of 56 women in this point prevalence cross-sectional study. The sample size was determined on the basis of the number of women attending the antenatal OPD (n-10000), the RPL prevalence in the general population (1%), and the Confidence Interval (5%). The sample size was calculated by the EPI Info software. The sample size was calculated as 38.

A history of RPL was elicited from all pregnant women attending the antenatal clinic at a large tertiary care hospital. The women who gave a history of RPL were further investigated and an attempt was made to establish the cause for the RPL. The pregnant women were then classified based on the inclusion and exclusion criteria and were grouped into two groups; Group A patients were the patients who conformed to the criteria for RPL, and group B patients were all pregnant women with a history of at least one successful pregnancy and with no previous history of a pregnancy loss or any other medical disorder.

The Polymerase Chain Reaction (PCR) followed by Restriction Fragment Length Polymorphism (RFLP) analysis was used to test for the G20210A mutation. This method has been described previously (4). The study was approved by the institutional ethics committee and informed consent was taken from all the patients. Data collected in this study was analyzed in Microsoft Excel and Stata v. 13 (Stata Corp, Texas, USA). The comparison of the two groups' means was done using two sample *t*-test with equal variances p < 0.05 was considered significant.

Of a total of 109 pregnant women who were recruited in the present study, 03 cases were excluded. PTG mutation was carried out in 38 women in Group A patients and 18 Group B patients. None of the group A patients were positive for PTG mutations, while one of eighteen women in Group B had heterozygous PTG mutation. The difference was not significant (p-0.24, Odds Ratio-0.1649, Confidence Interval-95%). It is believed that pregnancy is a physiological hypercoagulable state with an upregulation of Factor VIII and a downregulation of Protein S. The reasons for this hypercoagulable state is probably to help in the expulsion of the placenta with a minimal loss of blood. The Factor V Leiden mutation and the Prothrombin gene mutation are known to compound the hypercoagulable state.

PTG20210A mutation is seen in around 2-3% of the Caucasian population with estimated thrombotic risk being very low at 1.5-2.2 (5). Prothrombin mutation is a functional gain mutation occurring on exon 14 coding for 3' translational end of mRNA resulting in increased levels of Prothrombin.

Studies evaluating the role of Prothrombin gene mutations in cases of RPL have shown conflicting results. As reported in an Iranian study, there was no significant difference between the prevalence of these mutations between the cases versus the controls. (6). A similar finding was reported by Mierla *et al* who did not find an association between the PTG20210A mutations with RPL (7). This is in contrast to a Turkish study which found a significant association between the PTG20210A mutation and RPL (8).

The association between the PTG20210A mutation and RPL likely reflects the prevalence of this mutation in the general population. In Indian populations, the prevalence of the PTG20210A mutation has been evaluated in diseases as diverse as ischemic stroke and hepatic venous outflow tract obstruction, and the reported prevalence of this mutation is low (9). In a study of 432 patients of venous thromboembolism from western India, Ghosh *et al*. did not find a single case where there was a prothrombin PTG20210A mutation (10).

Of the total of 56 cases tested for Prothrombin mutation in the present study, no mutation was detected in RPL group (0/38), while one case was detected to have a heterozygous Prothrombin mutation in the control group (1/18).

We believe that this is the first Indian study which has studied the relationship between the Prothrombin gene mutation and RPL. It is likely that the prevalence of the PTG20210A gene mutation is very low in the Indian population. This low prevalence and its virtual absence in cases of RPL indicate that Prothrombin gene mutations are very unlikely to be a cause for RPL in the Indian population. It is suggested that an evaluation of the PTG is not required in cases of RPL in the Indian population.

**Ravindranath Reddy${}^{1}$ M.D., Deepti Mutreja${}^{1}$ M.D., Indrani Mukhopadhyay${}^{2}$ M.D., Nikhil Moorchung${}^{1}$ M.D., Ph.D.**

^1^Department of Pathology, Command Hospital Air Force, AGRAM Post, Cambridge Layout, Bangalore, India.

^2^Department of Gynaecology and Obstetrics, Command Hospital Air Force, AGRAM Post, Cambridge Layout, Bangalore, India.

## References

[1] (2008). *of the American Society for Reproductive Medicine. Definitions of infertility and recurrent pregnancy loss. Fertil Steril*.

[2] PRITCHARD A. M., HENDRIX P. W., PAIDAS M. J. (2016). Hereditary Thrombophilia and Recurrent Pregnancy Loss. *Clinical Obstetrics and Gynecology*.

[3] Settin A., Alkasem R. A., Ali E., ElBaz R., Mashaley A. M. (2013). Factor V Leiden and prothrombin gene mutations in Egyptian cases with unexplained recurrent pregnancy loss. *International Journal of Hematology*.

[4] Moussaoui S., Saussoy P., Ambroise J., Defour J. P., Zouitene R., Sifi K., Abadi N. (2016). Genetic Risk Factors of Venous Thromboembolism in the East Algerian Population. *Clinical and Applied Thrombosis/Hemostasis*.

[5] Chudej J., Plameňová I. (2016). Prothrombin gene 20210A mutation in Slovak population. *Vnitřní lékařství*.

[6] Poursadegh Zonouzi A., Chaparzadeh N., Ghorbian S., Sadaghiani M. M., Farzadi L., Ghasemzadeh A., Kafshdooz T., Sakhinia M., Sakhinia E. (2013). The association between thrombophilic gene mutations and recurrent pregnancy loss. *Journal of Assisted Reproduction and Genetics*.

[7] Mierla D., Szmal C., Neagos D., Cretu R., Stoian V., Jardan D. (2012). Association of prothrombin (A20210G) and factor V leiden (A506G) with recurrent pregnancy loss. Maedica (Buchar. *Jardan D. Association of prothrombin (A20210G) and factor V leiden (A506G) with recurrent pregnancy loss. Maedica (Buchar*.

[8] Isaoglu U., Ulug P., Delibas I. B., Yilmaz M., Kumtepe Y., Dogan H., Tasdemir S. (2014). The association between inherited thrombophilia and recurrent pregnancy loss in Turkish women. *Clinical and Experimental Obstetrics & Gynecology*.

[9] Munshi A., Kaul S., Aliya N., Shafi G., Alladi S., Jyothy A. (2009). Prothombin gene G20210A mutation is not a risk factor for ischemic stroke in a South Indian Hyderabadi Population. *Thrombosis Research*.

[10] Ghosh K., Shetty S., Madkaikar M., Pawar A., Nair S., Khare A., Pathare A., Jijina F., Mohanty D. (2001). Venous thromboembolism in young patients from Western India: a study. *Clinical and Applied Thrombosis/Hemostasis*.

